# Pauses as a Quantitative Measure of Linguistic Planning Challenges in Parkinson’s Disease

**DOI:** 10.3390/brainsci15111131

**Published:** 2025-10-22

**Authors:** Sara D’Ascanio, Fabrizio Piras, Caterina Spada, Clelia Pellicano, Federica Piras

**Affiliations:** Neuropsychiatry Laboratory, Department of Clinical Neuroscience and Neurorehabilitation, IRCCS Santa Lucia Foundation, Via Ardeatina 306, 00179 Rome, Italy; s.dascanio@hsantalucia.it (S.D.); c.spada@hsantalucia.it (C.S.); c.pellicano@hsantalucia.it (C.P.); federica.piras@hsantalucia.it (F.P.)

**Keywords:** Parkinson’s Disease, narrative production, discourse analysis, pausing patterns, linguistic planning

## Abstract

**Background/Objectives**: Pausing is a multifaceted phenomenon relevant to motor and cognitive disorders, particularly Parkinson’s Disease (PD). Thus, examining pauses as a metric for linguistic planning and motor speech difficulties in PD patients has gained significant attention. Here, we examined the production of silent and filled pauses (indexing difficulties at various linguistic processing levels) during narrative tasks to investigate the interplay between pausing behavior and informativeness/productivity measures. **Methods**: Individuals’ pausing patterns during narratives were analyzed relative to their syntactic context (within and between sentences expressing motor and non-motor related content), in 29 patients in the mild-to-moderate stage of PD, and 29 age-matched healthy speakers. The interaction between communicative metrics (informativeness and productivity), motor symptoms, cognitive capabilities, and pausing behavior was explored to characterize the mechanisms underlying pause production and its influence on discourse content. **Results**: PD patients’ pausing profile was characterized by an overall reduced number of pauses, longer silent pauses and fewer/shorter filled pauses, particularly before words that extend or specify the semantic content of sentences. Contrary to what was observed in healthy speakers, both the duration of silent pauses and the total number and duration of filled pauses could explain a significant proportion of variance in informativeness measures. Silent pause duration significantly correlated with measures of lexical access, indicating that cognitive processes influence pause production, while motor speech and cognitive challenges may also interact. **Conclusions**: Current results have significant implications for understanding discourse difficulties linked to PD and for formulating intervention strategies to improve communication efficacy.

## 1. Introduction

Parkinson’s disease (PD) is a neurodegenerative disorder characterized by a wide range of motor and non-motor symptoms related to dysfunctions in the dopaminergic circuit. While motor symptoms are commonly acknowledged, non-motor symptoms encompassing cognitive and speech-language deficits are less studied, even though impairments in effective communication are present in more than 90% of patients [1].

Current research indicates that limited communicative abilities in individuals with PD may arise from a complex interplay involving alterations in speech, language, and cognitive functions [2]. However, the precise relationship between these elements remains unclear [1,3]. The objective and quantitative assessment of communicative skills in spontaneous speech reveals that individuals with PD demonstrate a greater occurrence of grammatical, lexical, and verb tense errors/imprecisions when compared to subjects not affected by the condition [4,5,6,7]. Further, significant reductions in productivity measures and informativeness [6,8,9] have been documented, irrespective of cognitive deficits and speech-motor impairments. The typical motor dysfunctions observed in PD, such as hypokinesia or akinesia, can also impact communicative effectiveness. The most frequent motor speech disorder in the illness is dysarthria (mostly consisting of hypophonia and dysprosodia), characterized by short rushes of speech interrupted by prolonged periods of silence. However, heterogeneous speech rates in PD have also been reported [10], with faster rates due to articulatory difficulties and blurred contrasts between different speech sounds [11]. These disruptions in the temporal flow of discourse have the potential to affect the amount of information conveyed as they impact the listeners’ processing of speech and their representations of utterances [12].

An area that has attracted considerable interest is the examination of pauses (i.e., the absence of phonetic gestures or hesitation syllables) as a quantitative measure of linguistic planning challenges in individuals with PD [3,13,14,15]. Indeed, due to its multi-faceted nature, pausing may be particularly informative in a combined motor and cognitive disorder such as PD [2] as well as in other tauopathic parkinsonian syndromes characterized by rapid motor-cognitive deterioration [16]. As a matter of fact, pauses may reflect either the cognitive mechanisms underlying language production [5] (i.e., difficulties in the early stages of conceptualization and formulation), and problems during motor programming [17] and articulation [18,19,20] thereby providing significant insights into the relative impact of these dysfunctions on the observed challenges in natural speech formulation [3].

Actually, the production of non-lexical vocalizations [21] (filled pauses) may occur when the speaker is struggling with language formulation, reflecting a reaction to internal cues indicating that the linguistic construction process is not proceeding as planned [13]. Likewise, difficulties in formulating a message may result in silent pauses at unexpected locations or for extended durations, negatively impacting communication [12]. Previous evidence demonstrated that aside from the involvement of a motor component [4], the occurrence of more frequent and longer silent pauses at atypical locations (within a phrase or word) signifies the challenges faced by PD patients in the process of lexical retrieval [3,5,15,17], which may be particularly pronounced when the generation of verbs is required [3]. As silent pausing between utterances is assumed to provide time for the upcoming utterance [4], more frequent [5] or longer silent pauses at syntactic boundaries (as observable in PD [3,5,22]) are suggestive of a decline in semantic and syntactic planning/processing [3,4,5,22] and narrative organization [5]. However, although previous investigations conjunctly considered the intra- and inter-phrasal localization of pauses, therefore shedding light on their lexical/cognitive and syntactic function [3,5,22], the examination was limited to the production of silent pauses. In contrast, when both silent and filled pauses were contemplated, only the syntactic inter-phrasal level was explored [13], and insights regarding pausing as an indicator of linguistic conceptualization or formulation challenges were exclusively drawn from silent pauses [5,15,17].

Here, as a secondary analysis of narratives produced by patients diagnosed with PD and healthy comparators (HC) [9], pauses were evaluated as a quantitative measure of the linguistic construction challenges occurring during a naturalistic speech task. We indeed intended to assess the degree and nature of discourse planning difficulties (as revealed through individuals’ patterns of pausing [5]) and their relation to the previously documented decrease in productivity and informativeness in narratives [9].

Although the issue is debated, since PD patients have been reported to use fewer filled pauses, with an increase in silent pausing [13,23], alternatively showing a significant reduction of the total number of pauses compared to healthy speakers [24], we expect differential variations in the total number/duration of filled and silent pauses. Considering the role of basal ganglia in automatic, internally cued behaviors, we anticipate fewer/shorter filled pauses in the connected speech of PD patients [13] since these represent self-reparative mechanisms supporting speech in instances of error occurrence or when cognitive demands are heightened [25,26]. Concerning pause location, we anticipate a greater occurrence of (possibly silent) pauses at the lexical/semantic level (indexing difficulties in accessing semantic representations), particularly before verbs or within utterances conveying action content [3]. Such a finding is expected as it would account for the previously reported association between action naming and reduced productivity and informativeness measures in the connected speech of PD patients [9].

By concomitantly examining the production of both filled and silent pauses produced at different locations (either within and between sentences) and their potential association with several measures of discourse productivity and informativeness, we intended to provide preliminary evidence regarding the mechanisms responsible for the observed deficits in effective communication in individuals with PD. Our objective was indeed to ascertain whether local or global decision-making deficits (as indicated by prolonged silent pausing within and between utterances) adversely influenced the patients’ efficacy in conveying information. Furthermore, the simultaneous analysis of filled pauses would provide additional insight into the patients’ capacity to engage in self-repair mechanisms, as to potentially overcome the experienced challenges in discourse formulation and production.

## 2. Materials and Methods

### 2.1. Participants and Study Procedures

The present is a further analysis of narratives produced by patients diagnosed with PD and HC (see [9]). Participants and assessment procedures for neuropsychological/neuropsychiatric symptoms and for discourse analysis are detailed elsewhere [9]. Upon being informed about the study procedures (which were formally approved by the Ethics Committee of Santa Lucia Foundation through a written statement containing a waiver Prot. CE/PROG.905 20-01-21), each participant provided a signed informed consent for participating in the study and for the handling of their personal data. Information about data processing (including the secondary analysis herein reported) and about the potential publication of research results was included in the informed consent form.

Briefly, 29 patients with a confirmed PD diagnosis in the mild-to-moderate disease phase under stable antiparkinsonian medications, and 29 age- and gender-matched HC were enrolled in the study. Participants had normal cognitive status (scored > 24 on the Mini-Mental Status Examination [27,28] although they differed from HCs for neuropsychological performance, while presenting mild/moderate unipolar mood and/or anxiety disorders [29] and apathetic symptomatology [30] (see Table 1 for participants’ characteristics).

Subjects were required to describe a single picture image (“Cookie theft”, from the Boston Diagnostic Aphasia Examination [31]) and a cartoon story with six pictures (“Quarrel”, from [32]), to elicit natural speech as a proxy for longer narratives [33]. Stimuli were shown using a laptop turned toward the patient, and the examinee was asked to describe them as if the examiner did not know the story at all, with no further instructions or additional suggestions. Each storytelling was tape-recorded and subsequently transcribed verbatim by trained speech pathologists (SD and FeP) using the signal processing software Praat [34], to guarantee greater precision for the detection of silences, silent micro-pauses (>0.4 s) and hesitations. Such a threshold was selected to better capture pauses thought to reflect cognitive-linguistic, rather than speech motor processes [17]. As we examined the production of pauses both within and between utterances, a threshold capturing also longer pauses was considered adequate for the identification of silences that may signify local (i.e., within sentences) and distal (between utterances) challenges in lexical-semantic and syntactic decision-making processes [3,13,17]. The speech sample was further manually segmented into utterances according to semantic, grammatical and phonological criteria [35]. To explore whether pausing specifically differed in PD patients during action-related language production [3], verbs were manually identified and classified as action-verbs (describing a motor action or an intentional state) and non-action verbs (auxiliary or linking verbs), and utterances containing action-verbs were labeled as action-utterances (AU), while the remaining were considered non-action ones (non-AU) (see [3]). Sentences containing one action verb and one non-action verb were considered AU utterances because the content is centered on an action. First and second authors, blinded to participants’ group, independently analyzed transcripts for utterance segmentation and verb identification/classification. The inter-measurer comparisons revealed a general agreement of over 70%, with inconsistencies being addressed through discussions. The total number and duration of action and non-action utterances were computed (AU-N; AU-t; non-AU-N; non-AU-t).

### 2.2. Pauses Analysis

A silent pause was determined as any segment of non-speech longer than 400 ms [3]. Phonological fillers (“mmm”; “eeee”) and hesitations (“ehmm”) were counted as non-silent, filled pauses. Repetitions and lexical fillers (i.e., real words) were excluded from the filled pauses total count as these are automatic productions conveying semantic content [13]. The following measures of interest were then derived: the total number and duration of pauses (N-Ptot; T-Ptot) separately computed for silent (SP-N; SP-t) and filled pauses (FP-N; FP-t) and their mean duration (SP-DURMean; FP-DURMean).

Silent and filled pauses were then categorized according to their syntactic environment. Two levels of analysis (intra- and inter-sentence) [17] were considered, since, according to the existing literature, pauses before words represent lexical retrieval time and local lexical-semantic decision making, while pauses between utterances are indicative of difficulties in semantic-syntactic planning and narrative organization at the distal discourse level [36].

At the intra-sentence level, pauses were classified according to their position immediately before a noun, a verb (stratified as action/non-action) or other parts of speech (prepositions, adjectives, etc.) (single word level of analysis [15]). Pauses’ position within a sentence was also classified relative to typical prosodic boundaries, or atypical locations (grammatical intra-sentence level of analysis [5]). We then considered pause locations according to syntactic boundaries (inter-sentence or between-utterance level of analysis [5,17]) and defined whether they marked an independent clausal border (at the end of an independent clause and/or before a coordinated phrase) or a subordinate clausal boundary (before a finite or non-finite subordinate sentence).

The inter-measurer comparisons revealed a pause-by-pause agreement of over 70%, with inconsistencies being addressed through discussions. Then, the number and duration of pauses occurring before a noun (BN-N; BN-t), an action verb (BAV-N; BAV-t), a non-action verb (BnAV-N; BnAV-t), or other parts of speech (Baa-N; Baa-t) were separately computed for action and non-action utterances. At the same intra-sentence level, the number and duration of pauses occurring at typical (within a subordinate: WS-N; WS-t; between a subject and a predicate: BSP-N; BSP-t; in a listing: IL-N; IL-t) and atypical syntactic boundaries (between a pronoun and a verb: PV-N; PV-t, or within a word: WW-N; WW-t) [5,37] were separately calculated for AU and non-AU. Finally, at the inter-sentence level, the number and duration of pauses occurring after an independent finite clause (AI-N; AI-t) or between an independent and a subordinate clause (Bsub-N; Bsub-t) were computed. Table S1 reports a detailed description of pause categorization.

### 2.3. Narrative Language Outcomes

The current study exclusively considered indices that significantly differentiated patients from HCs (according to a previously performed multi-level discourse analysis [9]). A quantitative textual evaluation focusing on four main levels of discourse structure indeed demonstrated significant reductions in PD patients on measures of productivity (fewer well-formed words, shorter sentences) and informativeness (fewer conceptual units, less informative elements, lower number of details). However, articulation rate (number of syllables uttered divided by the total duration of the utterance, silent/filled pausing excluded) did not impact the observed reduced productivity and under-informativeness, which were rather explained by variations in linguistic abilities [9]. For the purpose of the present investigation and to control for variability in speaking rate, a new variable was computed as: modified word fluency = words divided by narrative time after subtracting the pausing time (WFmod) [3]. Additional language outcomes included: Narrative Time (NT-sec, considering talking time plus pauses), Total Words Count (TWC) and Mean Length of Utterances (MLU) as measures of productivity. The Percentage of Thematic Selection (%TH-S, the main ideas identified by speakers divided by the total number of the possible information elements), the total number of main concepts (words and verbs) accurately mentioned (CA-EssEA) and the total number of details (words and verbs) accurately stated (CA-DetEA) [38,39] were considered communicative informativeness indices (see Table 2 for language outcomes description).

### 2.4. Statistical Analyses

After having checked distribution normality (Shapiro-Wilk test), several language variables resulted in non-normally distributed data with considerable within-group variance. They were therefore logarithmically transformed, re-checked for normality and depending on results, general linear models or Mann–Whitney U-tests were used to analyze group differences on the variables of interest.

Non-parametric bivariate correlational analyses were conducted (separately in the two samples) between productivity and informativeness measures, differentiating the groups. Likewise, non-parametric correlation analyses examined the relationship between number and duration of filled/silent pauses and informativeness indices, with attention to potential variations according to pause location (intra- or inter-sentences). Additionally, in the patient group, non-parametric correlation analyses assessed the association between pausing variances and cognitive or motor symptoms. Bonferroni correction was applied to correlational analyses. All analyses were completed using IBM SPSS Statistics version 27.

## 3. Results

### 3.1. Productivity Indices and Their Relationship with Informativeness in the Studied Samples

Compared to HC, PD patients significantly produced fewer phonologically well-formed words (TWC, *p* = 0.01 and *p* = 0.02) and shorter sentences (MLU, *p* < 0.001) in both narrative tasks, although the number of uttered syllables in the narrative time did not affect the amount of produced words [9]. A multivariate analysis of variance with group membership (HC, PD) as an independent variable and the normalized NT-sec and modified WF_mod_ as dependent variables demonstrated a trend toward significance for the effect of group in both tasks (Wilk’s λ = 0.839; F_4,53_ = 2.538; *p* = 0.051; partial η^2^ = 0.161; power = 0.679). The univariate group effect was significant for NT-sec and not for WF_mod_. Between-subjects post-hoc pairwise comparisons revealed a decrease in NT-sec in both narratives among patients with PD, despite a speaking rate comparable to that of HC.

A series of non-parametric correlational analyses then explored (separately in the two groups) whether the previously observed reduced informativeness (PD < HC in %TH-S, CA-EssEA and CA-DetEA) [9] was related to the reported decreased productivity in PD. Bonferroni correction was applied as a *p*-value adjustment by multiplying each *p*-value by the number of included variables. A significant correlation (surviving to correction) was observable between narrative time and the number of details reported in the HC sample only, while in the PD group, not even uncorrected correlations were observable. Likewise, Bonferroni corrected correlations between the total number of well-formed words, the percentage of main ideas expressed and the total number of correctly stated essential elements and details were discernible in the HC sample, and not in the PD group (where correlations did not survive correction, p_corr_ > 0.05). Significant (after correction) correlations were also observed between another productivity measure (MLU, expressing proficiency in syntax and grammar) and the percentage of main ideas and details correctly reported for the cartoon story by HC speakers. The significant correlations between MLU and the percentage of main ideas and essential informative elements conveyed by PD patients did not survive correction for multiple comparisons. Table S2 reports results from the performed correlation analyses.

### 3.2. Pausing Parameters and Their Relationship with Informativeness Indices Differentiating the Two Groups

**Figure 1:**
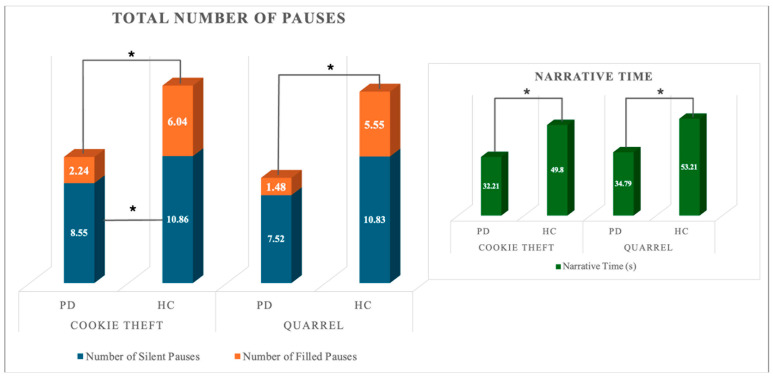
Quantitative pausing parameters and Narrative Time in the two groups. * indicate statistically significant differences.

**Figure 2:**
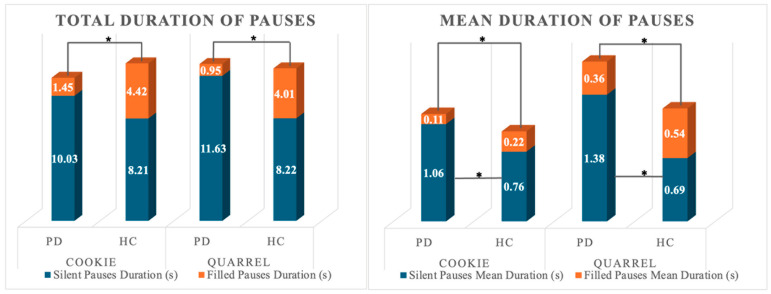
Total and Mean Duration of pausing in the two groups. * indicate statistically significant differences.

A significant multivariate effect of group (HC, PD) was observed on normalized pausing parameters in both narrative tasks (Wilk’s λ = 0.719; F_4,53_ = 5.176; *p* < 0.001; partial η^2^ = 0.281; power = 0.954 and Wilk’s λ = 0.497; F_4,53_ = 13.392; *p* < 0.001; partial η^2^ = 0.503; power = 1). The univariate effect of the group was significant for the total number of pauses and the total number of silent pauses in the complex picture description only. Between-subjects post-hoc pairwise comparisons on estimated marginal means revealed that PD patients produced a lower number of pauses overall and fewer instances of silent pauses compared to HCs. No other significant effect was observable. A Mann–Whitney U-test on total number and duration of filled pauses and mean duration of silent and filled pauses (FP-N; FP-t; SP-DUR_Mean_; FP-DUR_Mean_) revealed that HC and PD individuals differed for all the considered variables in both narrative tasks. PD patients showed a reduction in both the total number and average duration of filled pauses, whereas the average duration of silent pauses was prolonged in either task. Figure 1 depicts the significant differences between the total number of filled and silent pauses between groups. Differences in the total duration of active speech are also reported. Figure 2 reports the difference in total and mean duration of filled and silent pauses between groups.

Non-parametric correlational analyses showed significant correlations (not surviving to Bonferroni correction) between the total number of silent pauses and the number of correctly reported essential elements and details in the HC sample only. No significant results (not even at uncorrected *p*-values) were detectable in the PD sample. Likewise, the significant negative correlation between the mean duration of silent pauses and the percentage of main ideas expressed in the HC sample did not survive correction for multiple comparisons. Likewise, the correlation between the total number of produced pauses and the number of properly stated details in the HC sample did not survive correction. Of relevance, the mean duration of silent pauses was negatively related in the PD sample to the number of essential elements correctly conveyed, and such correlation survived Bonferroni correction. Pauses (and specifically silent ones) contributed to explaining the reduced informativeness in referential narratives (26% of the observed variance in the number of essential elements reported). See Table S2 for results from the performed correlational analyses.

### 3.3. Differences in Pausing Parameters According to Their Location. Association with Informativeness Indices, Neuropsychological Performance and Motor Symptoms

A Mann Whitney U-test on the duration of within sentences silent pauses (separately considering AU and non-AU, with occurrences N > 0) revealed no significant differences (according to location) in the complex picture description, while the total duration of silent pauses produced in non-AUs (PD > HC), the duration of silent pauses produced before a non-AV within AUs (PD < HC) and the duration of silent pauses before other parts of speech within non-AUs (PD > HC) significantly differed in the cartoon story narrative. The same analysis on the duration of the silent pauses between sentences demonstrated that PD patients produced significantly shorter pauses between an independent and a subordinate action clause.

Thus, although the number of non-AU silent pauses was the same in the two groups, PD patients produced longer silent pauses in general, and particularly before variant and invariant parts of speech such as prepositions, adjectives, etc., within sentences expressing non-action related content. Though the correlation between the latter and informativeness indices did not survive to Bonferroni correction (p_uncorr_ = 0.014), the duration of silent pauses preceding linguistic components that elaborate on the sentence’s content may elucidate 20% of the variability noted in the quantity of actions and elements that communicate the essence of the cartoon narrative. On the other hand, despite a comparable total duration of silent pauses produced within AUs, these were shorter in the PD patients’ sample when they occurred before a non-AV and a subordinate action clause. No pauses were observable at unexpected atypical syntactic boundaries in the patients’ group.

Table S3 reports the mean duration (in seconds) of intra- and inter-sentences pauses produced by the two groups and results from the performed analyses.

Separate non-parametric correlation analyses between silent pauses and the total number of crucial elements and accurately recounted details in the cartoon story showed no significant correlations (not even at an uncorrected level) in the HC group, while a trend toward a significant negative correlation was reported in PD patients between the duration of silent pauses produced before other parts of speech and the number of essential elements and actions correctly reported.  

Finally, in order to explore the potential association between language pausing, cognitive abilities and motor symptom in the PD sample, non-parametric correlations were run considering SP-DUR_Mean_ in the complex picture description, SP-Baa-non-AU-t in the cartoon story narrative (the pausing parameters explaining variance in informativeness measures), raw scores in neuropsychological tests indexing executive functioning, the patients’ semantic-lexical competence and the severity of motor symptoms (see [9] for cognitive assessment procedures). A significant negative correlation (surviving to correction for multiple comparisons) was observable between pauses before other parts of speech and action naming performance, while the negative correlation between pauses mean duration and object naming capacity did not survive to correction. Likewise, the positive correlation between the former and the severity of motor symptoms was only marginally significant after correction. Table S4 includes results from the correlation analyses between pausing parameters, cognitive abilities and motor symptoms.

**Figure 3:**
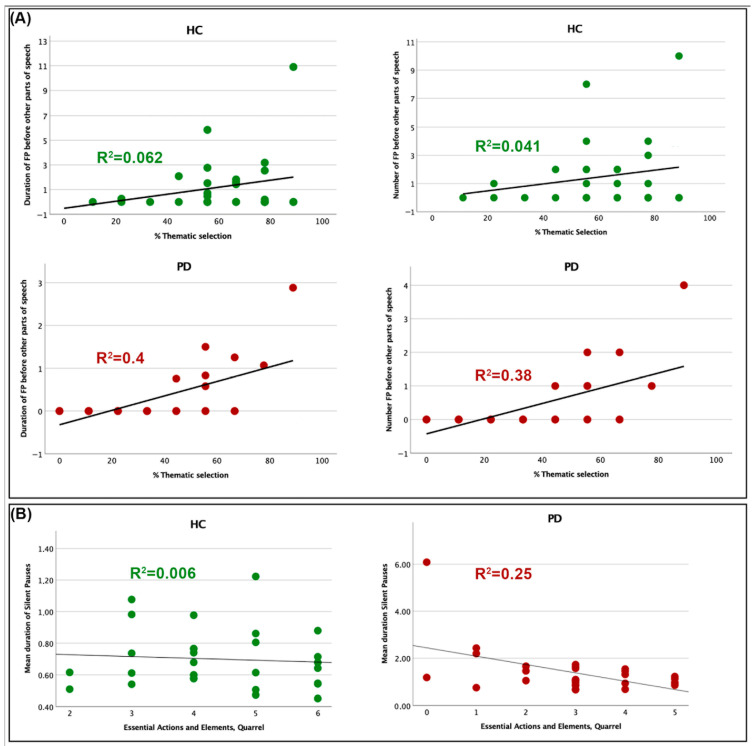
Correlations between total and mean duration of filled and silent pauses and informativeness indices (**A**) Relationships between the number and duration of filled pauses at specific discourse planning points and the percentage of main ideas expressed in the two groups. (**B**) Correlations between the mean duration of silent pauses and the number of essential elements reported in the two groups.

As ancillary analyses, separate non-parametric correlations between filled pausing parameters significantly differentiating the two groups and informativeness indices demonstrated a significant (Bonferroni corrected) correlation in the PD group, between the number and duration of filled pauses occurring before other parts of speech in AU sentences and the proportion of thematic elements produced in the complex picture description (explained variance 37 and 38% respectively). Such findings would suggest that when PD patients do generate filled pauses at these particular cognitive planning junctures, they demonstrate a markedly enhanced capacity to articulate the principal narrative concepts. Significant correlations between % TH-S and the number and duration of within-subordinate filled pauses did not survive correction. No significant correlations (not even uncorrected) were observable between the same filled pausing and informativeness parameters in the HC group. However, significant correlations (not surviving to correction) were discernible between the number and duration of within-subordinate filled pauses in AU sentences and the number of details correctly reported in the cartoon story by HC. Figure 3 depicts the observed significant correlations between the number and duration of filled pauses at specific discourse planning points and the percentage of main ideas expressed in the two groups. The correlation between the mean duration of silent pauses and the number of essential elements correctly conveyed in the two groups is also depicted.

The potential associations between filled pausing parameters contributing to explain informativeness variance in the PD group and neuropsychological performance in executive functioning and language measures, and the severity of motor symptoms were also tested. No significant correlation (not even at an uncorrected level of significance) was observable. Results are reported in Table S4.

## 4. Discussion

Here, we intended to explore whether pauses during spoken narratives could be regarded as valid measures of PD patients’ difficulties in language formulation and production [3,5]. We assumed that connected speech in the disorder would be characterized by a specific pausing pattern. To probe our assumptions, pauses indexing difficulties in lexical retrieval and semantic choices (i.e., those produced within sentences) or syntactic planning/discourse organization (i.e., occurring between utterances) were separately analyzed. The differential occurrence of pauses in action versus non-action sentences was also examined, considering verb use impairment in PD patients [3,40,41,42] and the documented decline in the spontaneous generation of action verbs as the symptoms of the disease advance [43]. Correlational analyses assessed whether pauses indicative of local (within utterances) or global (between) linguistic decision-making deficits accounted for reduced discourse fluency and informativeness [9]. The correlation between pausing parameters that differentiated groups and PD patients’ cognitive and motor dysfunctions was evaluated to determine their influence on pausing behavior [3,8,15].

We found that while in healthy comparators, measures of linguistic productivity could explain a significant proportion of observed variance in informativeness, PD patients were less informative, irrespective of their productivity. Their narrative time was shorter, despite a comparable speaking rate; they produced fewer words and more concise sentences, though these variables were not related to efficiency in spoken discourse. Moving forward, we found that compared to healthy speakers, PD patients produced fewer pauses (either silent or filled). The general reduction of verbal output did not account for the observed decrease, as when considered together, the ratio among pausing parameters, the quantity of phonologically well-formed words generated, and the aggregate duration of active speech (excluding silent and filled pauses) was the same across both cohorts. Nevertheless, patients with Parkinson’s disease exhibited an average of 3 filled pauses per 100 spoken words (in contrast to 5 within the HC sample) and 12 silent pauses per 100 words (compared to 9 in the HC group), with this discrepancy achieving statistical significance (*p* = 0.03 and *p* = 0.04). The ratio of silent pauses to the duration of active speech was calculated to be 0.60 in PD patients (0.21 in the healthy control sample, *p* < 0.001), thereby indicating a dysfluent speech production characterized by protracted silences. Crucially, the average duration of silences could explain 26% of observed variance in the number of correctly stated essential elements and actions in narratives. Contrariwise, pauses produced by healthy comparators were not significantly related to informativeness indices. This would suggest that extended silent pausing [13] may reflect inefficiencies in language formulation, potentially linked to challenges in cognitive processes such as word retrieval, monitoring and planning [44]. Extended silent pausing was, however, coupled with fewer and shorter filled pauses (accounting for a small portion of active speech: 0.1 compared to the 0.23 observed in HC; *p* = 0.03-), confirming that automatic responses to speech difficulties (i.e., the production of non-lexical vocalizations [21]) are impaired in PD patients [13]. Indeed, it has been demonstrated that filled pausing, providing temporal resources for rectifying errors and inconsistencies [21], is reduced in individuals with PD due to challenges in utilizing internal cues for behavioral modulation [26]. Decreases in dopamine levels in the disorder impede the ability of both external and internal stimuli to elicit an automatic response, and PD patients appear to depend more significantly on external stimuli to facilitate necessary adjustments [13]. Since the neural pathways responsible for the transmission of internal cues are particularly vulnerable to the initial spread of the disease [45], a reduction in filled pausing may be evident since the early stages (as in the present sample) and become increasingly noticeable as the disease progresses. Thus, from a clinical perspective, a diminution in filled pauses may function as a preliminary indicator of disease, also aiding in the assessment of illness trajectory [46]. Nevertheless, when specifically probed, no significant correlation was observable between pausing parameters differentiating the two groups and dopamine replacement treatment, suggesting that alternative neurotransmitter systems may be implicated in the characterization of patients’ pausing profile. Considering pause location (as an index of specific language formulation difficulties [5]), patients exhibited shorter silent and filled pauses in action-related utterances, especially before non-motor action verbs. In contrast, non-action utterances, prevalent in the patient group, contained longer silent pauses before elements other than verbs and nouns, while filled pauses were shorter before such elements in action utterances. This observation would suggest that silent pauses marked a potential impairment of the mechanism involved in constructing a syntactically well-formed sentence [4] since their duration was extended before words describing, defining or changing the information given by a noun or a verb (i.e., adjectives, determinants, adverbs, prepositions, etc.). The aggregated duration of silent pauses before these linguistic elements accounted for 20% of the variance in essential elements reported, indicating decreased informativeness with increased duration. Conversely, the number and duration of filled pauses before elements other than verbs and nouns positively correlated with the thematic elements produced, explaining over one-third of variance, suggesting that PD patients may benefit from the allocation of additional time for speech planning and execution, enhancing informativeness. Thus, we may speculate that while silent pausing marked the disruption of cognitive mechanisms for sentence planning/construction, filled pauses represented self-reparative behaviors to overcome the experienced challenges in linguistic conceptualization or formulation [47].

We might therefore suppose that PD patients’ pausing profile (longer silent pauses and fewer/shorter filled pauses) denotes difficulties in conceptualization and self-repair during speech production [47], contributing to explaining the observed reduced informativeness of narratives. This hypothesis is reinforced by the only previous study concurrently exploring the production of silent and filled pauses in the disorder [13], reporting a greater duration for silent pauses and fewer filled ones. The authors suggested that changes in PD patients’ speech and communication may arise from both difficulties in speech production and alterations in the capacity to automatically adapt in response to these challenges. Likewise, more frequent and longer silent pausing within utterances was interpreted as a difficulty in planning and preparing for sentences [5], which correlated with cognitive functioning [3], also potentially reflecting delays in the fluent transition between words (a form of “speech freezing” [17,48]). Actually, although produced in the context of non-AU, longer silent pauses positively correlated with action verb naming ability. However, confrontational naming tests may not reflect the complexities of continuous speech [15], and contrary to earlier studies [3], silent pauses were not more frequent before action verbs. Additionally, a marginally significant positive correlation was found between silent pause duration and motor symptom severity, indicating that a potential delay in motor program transitions may influence pausing behavior [17]. Nevertheless, patients did not exhibit pauses at inappropriate linguistic boundaries, a behavior previously linked to motor speech difficulties exacerbated by syntactic processing impairments [37]. Additionally, the absence of differences in speech motor function between PD patients and healthy comparators casts doubt on the theory that motor impairments solely influence pausing behavior. These observations would impede the possibility of clarifying the cognitive or motor nature of the reported pausing behavior, suggesting that a combination of motor impairments in speech production and cognitive/linguistic difficulties [37] may more adequately account for the observed pausing profile in PD and for the resultant decrease in productivity and informativeness. However, interventions to enhance motor system support for speech did not yield significant improvements in semantic, syntactic, or informative aspects of spoken discourse [49]. Concurrently, improvements in motor function after aerobic training led to enhanced sentence completeness in picture description tasks, without notable changes in fluency or grammaticality [50]. This would suggest that the observed differences in pausing behavior and the reduced informativeness in PD should be specifically targeted through rehabilitative interventions aimed at sustaining the later stages of utterance planning. For example, since extended pausing right before meaning-expanding elements advocates for syntactical difficulties in PD patients’ narratives (which were less detailed and informative), language interventions for expanding the repertoire of grammatical structure of sentences [51] might have a positive impact on overall communicative efficiency. Alternatively, treatments like the Verb Network Strengthening (VNeST) [52] may be suitable to augment informativeness in the disorder, given the reported association between pauses indicative of challenges in sentence expansion and the capacity to retrieve verb representations. However, these observations are fundamentally conjectural and based on inferences, as the current investigation did not implement any intervention. Nonetheless, in light of the detrimental effects of speech and language disorders on the quality of life of individuals with PD, coupled with the significance of expository narrative competencies for achieving success in social, academic, and professional domains, subsequent research should elucidate the ramifications of inappropriate pausing on communicative efficacy.

Before some concluding remarks, a few limitations of the present investigation should be acknowledged. First, the threshold employed here for identifying silences (>400 ms) may be regarded as questionable since previous studies have used different cut-offs to discriminate pauses with lexical/cognitive-linguistic function within utterances (e.g., >250 ms [22]). However, a former investigation has revealed data-driven categorization of silences as articulatory (around <100 ms), and longer pauses rooted in lexical retrieval, prosodic and/or syntactic purposes [17]. Thus, as we intended to investigate the linguistic function of pauses occurring both intra- and inter-utterance, a threshold capturing longer silences was deemed adequate for the identification of pauses signifying local and distal challenges in lexical-semantic and syntactic decision-making processes. Nevertheless, although quantitative results were the same when a coarser threshold was used (< and >1 s), future studies should verify whether alternative cut-offs may better characterize the PD patients’ pausing profile. One next step would also be to consider the relationship between filled and silent pauses, as PD patients have been reported to mark” longer silent pauses with filled ones [13]. A similar finding would have reinforced our hypothesis that the ability to automatically respond to speech formulation difficulties may be impaired in PD patients (as they produced fewer filled pauses), and the number and duration of silent and filled pauses occurring simultaneously will be computed in future analyses.

The relatively small sample size may constitute an additional limitation of the present investigation. However, post-hoc evaluations indicated a low probability of erroneously concluding that silent pause duration significantly influenced informativeness (0.13, Power = 0.87), while the power for the relationship between silent pauses before words other than nouns and verbs and informativeness was below the optimal threshold (0.36, Power = 0.74), slightly diminishing confidence in the findings.

Nonetheless, consistent with previous conclusions [4], we suggested that difficulties in constructing detailed compound sentences, characterized by prolonged within-utterance silent pauses, may account for the reduced conveyance of essential narrative elements. Indeed, quantitative measures of the specular mechanism (the production of longer filled pauses to overcome the experienced challenge) were related to patients’ informativeness, and the achieved statistical power (0.98) supports our hypothesis. Nevertheless, the present findings may hold true only for the akinetic-rigid motor subtype of PD (70% of the investigated patients), while we were not able to further characterize our sample, considering more comprehensive and neurobiological subtypes. Indeed, previous evidence [53] demonstrated in de novo PD patients a different speech profile in the postural instability/gait disorder motor subtype (as compared to the tremor-dominant), suggesting that the observed speech rhythm disorder was subtended by pathomechanisms specific to this subgroup. Future studies using a more comprehensive characterization of PD’s subtypes will help clarify the issue.

## 5. Conclusions

To our knowledge, this is the first study quantitatively examining the production of pauses indexing difficulties at various tiers of linguistic processing (i.e., both silent and filled pauses), while also considering their syntactic contexts (within and across sentences), as well as the possible implications for discourse informativeness. By characterizing the pausing profile of PD patients during an ecologically valid measure of expressive language, we were able to demonstrate that difficulties in linguistic planning, and particularly in accessing lexical items that extend or specify the semantic content of sentences, exhibited a negative correlation with the patients’ capacity to effectively convey pertinent information. Concomitantly, self-repair mechanisms (such as the production of non-lexical vocalizations and hesitations) were positively related to the number of main ideas conveyed, thereby suggesting that the allocation of additional temporal resources for the planning and formulation of speech may represent a viable strategy for enhancing PD patients’ informativeness.

From a clinical perspective, the present results bear important implications as they highlight the fact that simple non-invasive speech and language assessments have the potential to capture subtle cognitive/linguistic disturbances even in the early stage of the disease. Crucially, recent research highlighted the significance of objective linguistic indicators in detecting PD in the initial (i.e., before motor symptoms onset) or even prodromal stages [54], implicating a potential window for timely pharmacological treatment [46]. Thus, changes in pausing profile, like the here observed reduction in filled pauses, may serve as an initial marker of pathology, facilitate the evaluation of the progression of illness, and additionally inform subsequent treatment strategies. For example, the provision of interventions aimed at improving self-repair by augmenting self-monitoring (through parsing of inner or overt speech) [25] and the production of editing terms or phrases (to maintain the conversational turn) [55] may increase the probability of producing a well-formed repair. This would potentially enhance patients’ ability to efficiently communicate new and relevant content, with important implications on the quality of social interactions and life contentment.

Although preliminary, considering the above-mentioned limitations and the inherently correlational design of the study, we posit that our findings shed further light on the discourse difficulties observable in PD patients. Recent advancements in automatic discourse processing have the potential to facilitate cost-efficient and scalable monitoring across larger cohorts, thereby ultimately enabling the incorporation of speech and language biomarkers into clinical protocols for screening, diagnosing, subtyping, and monitoring PD. The methodology employed herein, which investigates location-specific occurrences of both silent and filled pauses in relation to the informative content, aspires to contribute to the expansion of the research domain.

## Figures and Tables

**Table 1:** Mean sociodemographic, clinical, psychopathological, cognitive and linguistic characteristics of the studied samples.

Characteristics (Standard Deviation)	HC (n = 29)	PD (n = 29)	t or χ2	*d.f.*	*p*
Age (years/sd)	62.21 (9.43)	63.00 (8.46)	−0.34	56	0.74
Males n (%)	16 (55)	18 (62)	0.28	1	0.59
Educational level (years/sd)	13.17 (3.64)	11.24 (3.85)	1.96	56	**0.05 ^§^**
Duration of illness (years/sd)	-	4.02 (2.61)	-	-	-
Modified H&Y score	-	1.83 (0.54)	-	-	-
MDS-UPDRS-III score (sd)	-	14.28 (8.61)	-	-	-
Levodopa equivalents (mg/day-sd)	-	477.58 (259.41)	-	-	-
PPRS (score/sd)	-	6.72 (1.07)	-	-	-
AS tot. (score/sd)	2.31 (2.22)	4.38 (4.79)	−2.24	39.80	0.03 *
HAMA tot. (score/sd)	4.62 (3.76)	6.31 (4.86)	−1.31	56	0.19
BDI tot. (score/sd)	5.10 (4.06)	7.52 (6.12)	−1.68	56	0.09
MMSE (raw score/sd)	29.52 (0.63)	29.10 (1.08)	1.78	45.22	0.08
WCST-PE	0.31 (0.81)	1.97 (3.27)	−2.65	31.39	0.01 *
SWCT-IE-T (sec/sd)	35.86 (9.74)	41.83 (21.85)	−1.34	38.71	0.19
RF-C	32.28 (2.84)	29.07 (5.71)	2.71	41.07	0.01 *
SW-T	43.97 (10.15)	37.66 (11.81)	2.18	54.76	0.03 *
Ph-F	40.48 (10.23)	34.52 (9.92)	2.25	56	0.03 *
Sem-F	24.34 (4.78)	21.41 (5.99)	2.06	53.38	0.04 *
Ac-N (27 subjects)	-	53.74 (5.08)	-	-	-
Ob-N (27 subjects)	-	56.33 (4.27)	-	-	-

Legend: Ac-N, Action Naming; AS, Apathy Scale; BDI, Beck Depression Inventory; d.f., degree of freedom; HC, healthy controls; Modified H&Y, Hoehn and Yahr scale; HAMA, Hamilton Anxiety Rating Scale; HC, healthy controls; WCST-PE, Wisconsin Card Sorting Test perseverative errors; MMSE, Mini-Mental State Examination; Ob-N, Object Naming; PD, Parkinson Disease patients; Ph-F, phonological fluency; PPRS, Parkinson’s Psychosis Rating Scale; RF-C, Ray Figure copy; Sem-F, semantic fluency; SW-T; total number of switches in fluency tasks; SWCT-IE-T, Stroop Word-Color Test interference effect time; MDS-UPDRS-III scale, Movement Disorder Society-Sponsored Revision of the Unified Parkinson’s Disease Rating Scale Part III motor function. Bold and * indicate statistically significant differences. § indicates trend to significance.

**Table 2:** Language outcomes description.

PRODUCTIVITY	
Modified word fluency (WF_mod_)	Words are divided by narrative time (NT) after subtracting the pausing time
Total word count (TWC)	The total number of phonologically well-formed words excluding phonological fillers, phonological errors and nonwords
Mean Length of Utterances (MLU)	The total number of phonologically well-formed words divided by the number of utterances produced
INFORMATIVENESS	
Percentage of thematic selection (% TH-S)	The thematic selection encompassed all the principal concepts recognized by the speaker within the story. This was achieved by calculating the ratio of the total number of main ideas articulated in each picture description to the overall number of possible information components. The anticipated quantity of content units was predetermined through the analysis of the control group’s performance, identifying two fundamental categories of information: target contentive words or thematic units crucial for grasping the essence of the story, and additional relevant content units that relay supplementary, non-essential information
Essential action and elements (CA-EssEA)	The total number of main concepts (words and verbs) accurately mentioned
Actions and elements details (CA-DetEA)	The total number of details (words and verbs) accurately stated

## Data Availability

The data supporting the findings of this study are available on request from the corresponding author. The data are not publicly available due to privacy/ethical restrictions.

## Supplementary Materials

The following supporting information can be downloaded at: https://www.mdpi.com/article/10.3390/brainsci15111131/s1, Table S1: Detailed description of pauses categorization; Table S2: Correlation analyses between productivity measures, pausing parameters and informativeness in the two groups; Table S3: Intra and inter-sentences pauses produced by the two groups and results from the performed analyses; Table S4: Correlation analyses between pausing parameters differentiating the two groups, cognitive abilities and motor symptoms in PD patients.
