# Supplementary material for: Pauses as a Quantitative Measure of Linguistic Planning Challenges in Parkinson’s Disease
Source: Brain Sci. 2025 Oct 22;15(11):1131. doi: 10.3390/brainsci15111131 (PMC12650203; doi:10.3390/brainsci15111131)
Supplement: Supplementary file 1 [file brainsci-15-01131-s001.zip › brainsci-3882738-supplementary.pdf]

Supplementary materials

S1 - Detailed description of pauses categorization.

			**SILENT PAUSES (SP)**		**FILLED PAUSES (FP)**	
			Action utterances (AU)	non-Action utterances (non-AU)	Action utterances (AU)	non-Action utterances (non-AU)
**INTRA-SENTENCE LEVEL OF ANALYSIS - WITHIN UTTERANCES**	**Single word level of analyses**	Before non-action verb	SP-BnAV-AU-N	SP-BnAV-non-AU-N	FP-BnAV-AU-N	FP-BnAV-non-AU-N
			SP-BnAV-AU-t	SP-BnAV-non-AU-t	FP-BnAV-AU-t	FP-BnAV-non-AU-t
		Before action verb	SP-BAV-AU-N	SP-BAV-non-AU-N	FP-BAV-AU-N	FP-BAV-non-AU-N
			SP-BAV-AU-t	SP-BAV-non-AU-t	FP-BAV-AU-t	FP-BAV-non-AU-t
		Before noun	SP-BN-AU-N	SP-BN-non-AU-N	FP-BN-AU-N	FP-BN-non-AU-N
			SP-BN-AU-t	SP-BN-non-AU-t	FP-BN-AU-t	FP-BN-non-AU-t
		Before other parts of speech	SP-Baa-AU-N	SP-Baa-non-AU-N	FP-Baa-AU-N	FP-Baa-non-AU-N
			SP-Baa-AU-t	SP-Baa-non-AU-t	FP-Baa-AU-t	FP-Baa-non-AU-t
	**Grammatical level of analyses**	Within subordinate	SP-WS-AU-N	SP-WS-non-AU-N	FP-BWS-AU-N	FP-WS-non-AU-N
			SP-WS-AU-t	SP-BWS-non-AU-t	FP-WS-AU-t	FP-WS-non-AU-t
		Between subject and predicate	SP-BSP-AU-N	SP-BSP-non-AU-N	FP-BSP-AU-N	FP-BSP-non-AU-N
			SP-BSP-AU-t	SP-BSP-non-AU-t	FP-BSP-AU-t	FP-BSP-non-AU-t
		In a listing	SP-IL-AU-N	SP-IL-non-AU-N	FP-IL-AU-N	FP-IL-non-AU-N
			SP-IL-AU-t	SP-IL-non-AU-t	FP-IL-AU-t	FP-IL-non-AU-t
		Between pronoun and verb	SP-PV-AU-N	SP-PV-non-AU-N	FP-PV-AU-N	FP-PV-non-AU-N
			SP-PV-AU-t	SP-PV-non-AU-t	FP-PV-AU-t	FP-PV-non-AU-t
		Within word	SP-WW-AU-N	SP-WW-non-AU-N	FP-WW-AU-N	FP-WW-non-AU-N
			SP-WW-AU-t	SP-WW-non-AU-t	FP-WW-AU-t	FP-WW-non-AU-t
**INTER-SENTENCE LEVEL OF ANALYSIS-BETWEEN UTTERANCES**	**Syntactic level of analyses**	After independent	SP-AI-AU-N	SP-AI-non-AU-N	FP-AI-AU-N	FP-AI-non-AU-N
			SP-AI-AU-t	SP-AI-non-AU-t	FP-AI-AU-t	FP-AI-non-AU-t
		After independent and before subordinate	SP-BSub-AU-N	SP-BSub-non-AU-N	FP-BSub-AU-N	FP-BSub-non-AU-N
			SP-BSub-AU-t	SP-BSub-non-AU-t	FP-BSub-AU-t	FP-BSub-non-AU-t

Legend: N: number of occurrences; t: duration.

S2 - Correlation analyses between productivity measures, pausing parameters and informativeness in the two groups.

COOKIE THEFT												
	**%TH-S**				**CA-EssEA**				**CA-DetEA**			
	**HC**		**PD**		**HC**		**PD**		**HC**		**PD**	
	**ϱ**	**p**	**ϱ**	**p**	**ϱ**	**p**	**ϱ**	**p**	**ϱ**	**p**	**ϱ**	**p**
NT-sec	0.419	0.024*	0.231	0.228	0.465	0.011*	0.01	0.958	0.368	0.05*	-0.020	0.919
TWC	0.561	**0.002****	0.424	0.022*	0.515	**0.004****	0.239	0.211	0.353	0.06	0.054	0.781
MLU	0.313	0.098	0.416	0.025*	0.043	0.826	0.446	0.015*	-0.103	0.594	0.244	0.202
N-P_{tot}	0.139	0.472	0.156	0.418	0.33	0.081	-0.065	0.737	0.352	0.061	-0.093	0.630
FP-N	0.048	0.805	0.234	0.222	0.192	0.317	-0.033	0.863	0.171	0.375	-0.142	0.463
FP-t	0.091	0.638	0.293	0.122	0.172	0.371	0.04	0.838	0.152	0.43	-0.115	0.553
SP-N	0.24	0.21	0.168	0.384	0.415	0.025*	-0.026	0.892	0.417	0.024*	-0.062	0.749
SP-DUR_{Mean}	-0.379	0.043*	-0.263	0.168	-0.137	0.478	-0.321	0.09	0.096	0.621	-0.101	0.601
FP-DUR_{Mean}	-0.037	0.847	0.254	0.184	0.009	0.963	-0.009	0.962	-0.044	0.822	-0.071	0.716

**QUARREL**												
	**%TH-S**				**CA-EssEA**				**CA-DetEA**			
	**HC**		**PD**		**HC**		**PD**		**HC**		**PD**	
	**ρ**	**p**	**ρ**	**p**	**ρ**	**p**	**ρ**	**p**	**ρ**	**p**	**ρ**	**p**
NT-sec	0.442	0.016*	0.106	0.584	0.172	0.372	-0.015	0.937	0.563	**0.001****	0.108	0.578
TWC	0.659	**0.000****	0.412	0.026*	0.336	0.075	0.268	0.16	0.691	**0.000****	0.302	0.111
MLU	0.522	**0.004****	0.401	0.031*	0.383	0.04*	0.359	0.056	0.549	**0.002****	0.346	0.066
N-P_{tot}	-	-	-	-	0.154	0.426	-0.004	0.985	0.384	0.04*	0.009	0.962
FP-N	-	-	-	-	0.153	0.428	0.064	0.742	0.326	0.085	0.081	0.676
FP-t	-	-	-	-	0.153	0.428	0.049	0.799	0.296	0.12	0.106	0.584
SP-N	-	-	-	-	-	-	-	-	-	-	-	-
SP-DUR_{Mean}	-	-	-	-	-0.077	0.693	-0.508	**0.005****	0.098	0.612	-0.162	0.400
FP-DUR_{Mean}	-	-	-	-	0.227	0.235	0.064	0.743	0.083	0.667	0.041	0.833

Legend: CA-EssEA - Essential action and elements; CA-DetEA - Actions and elements details; FP-DUR_{Mean} - Mean duration of filled pauses; FP-N - Total number of filled pauses; FP-t - Total duration of filled pauses; HC, healthy controls; MLU – Mean Length of Utterance; N-P_{tot} - Total number of pauses; NT-sec - Narrative Time (talking time plus pauses); PD, Parkinson Disease patients; SP-DUR_{Mean} - Mean duration of silent pauses; TWC – Total word count; %TH-S – Percentage of thematic selection. **Uncorrected significance (correlation surviving after Bonferroni correction, highlighted in bold); *Uncorrected significance (correlation not surviving after Bonferroni correction).

S3 - Intra and inter-sentences pauses produced by the two groups and results from the performed analyses.

	COOKIE THEFT				QUARREL			
	**HC (29)**	**PD (29)**	**U**	**p**	**HC (29)**	**PD (29)**	**U**	**p**
SP-AU-t	6.25 (3.39)	6.31 (4.69)	402.00	0.77	7.15 (7.64)	7.90 (7.10)	490	0.28
SP-non-AU-t	1.98 (2.41)	3.78 (8.56)	402	0.763	1.08 (3.00)	3.67 (7.75)	558	**0.024***
FP-AU-N	4.86 (6.36)	1.90 (2.61)	292	**0.042***	5.07 (9.94)	1.17 (2.42)	241.5	**0.004***
FP-AU-t	3.47 (5.23)	1.24 (1.86)	285	**0.033***	3.64 (7.48)	0.72 (1.46)	244	**0.005***
FP-non-AU-N	1.21 (1.52)	0.31 (0.66)	250.5	**0.003***	-	-	-	-
FP-non-AU-t	0.97 (1.15)	0.21 (0.45)	242	**0.002***	-	-	-	-
SP Intra-sentences								
SP-BN-AU-t	1.67 (1.12)	2.02 (2.69)	360	0.345	1.91 (2.16)	2.09 (2.56)	416	0.943
SP-BN-non-AU-t	0.57 (1.01)	0.91 (2.47)	380	0.450	0.38 (0.84)	0.65 (1.11)	475	0.292
SP-BAV-AU-t	1.14 (1.19)	1.64 (1.48)	501	0.208	1.28 (1.46)	1.66 (1.45)	488.5	0.286
SP-BnAV-AU-t	0.80 (1.53)	0.45 (0.95)	334	0.128	0.97 (1.75)	0.28 (0.63)	314	**0.047***
SP-BnAV-non-AU-t	0.21 (0.53)	0.78 (1.66)	475	0.246	0.14 (0.38)	0.80 (3.07)	451.5	0.464
SP-Baa-AU-t	2.84 (2.00)	2.11 (1.83)	319	0.114	2.94 (3.71)	3.21 (6.98)	316.5	0.104
SP-Baa-non-AU-t	0.70 (1.19)	1.91 (6.08)	395	0.661	0.54 (2.17)	1.99 (4.62)	541	**0.025***
SP-WS-AU-t	1.75 (1.83)	1.58 (1.89)	377,5	0.497	1.12 (1.56)	0.93 (1.28)	372	0.436
SP-BSP-AU-t	0.33 (0.55)	0.36 (0.57)	427	0.906	0.21 (0.41)	0.35 (0.98)	365	0.237
SP-IL-AU-t	-	-	-	-	0.41 (1.15)	1.43 (3.24)	448	0.532
FP Intra-sentences								
FP-BnAV-AU-N	0.83 (1.44)	0.03 (0.19)	272.5	**0.001***	0.90 (2.23)	0.03 (0.19)	288.5	**0.003***
FP-BnAV-AU-t	0.62 (1.08)	0.02 (0.10)	272	**0.001***	0.60 (1.44)	0.03 (0.14)	289	**0.003***
FP-Baa-AU-N	1.48 (2.43)	0.41 (0.91)	302	**0.032***	2.14 (4.76)	0.24 (0.69)	287	**0.010***
FP-Baa-AU-t	1.22 (2.31)	0.31 (0.66)	305	**0.037***	1.57 (3.65)	0.09 (0.24)	278	**0.006***
FP-WS-AU-N	1.59 (2.26)	0.41 (0.78)	286	**0.018***	1.28 (2.60)	0.21 (0.77)	313.5	**0.023***
FP-WS-AU-t	1.05 (1.68)	0.28 (0.62)	292.5	**0.026***	0.97 (2.24)	0.12 (0.46)	313	**0.022***
SP Inter-sentences								
SP-AI-AU-t	2.18 (1.53)	2.22 (2.95)	354	0.301	3.18 (3.02)	5.29 (4.98)	530.5	0.087
SP-BSub-AU-t	0.47 (0.73)	0.59 (1.14)	405	0.787	0.39 (0.51)	0.19 (0.64)	298	**0.018***
SP-AI-non-AU-t	0.80 (1.33)	1.84 (3.96)	510.5	0.137	0.61 (1.46)	1.61 (3.47)	455	0.539

Legend: AI – after independent; AU – action utterance; Baa – before other parts of speech; BAV – before action verb; BN – before noun; BnAV – before non-action verb; BSP – between subject and predicate; Bsub – before subordinate; FP – filled pause; HC, healthy controls; IL – in a listing; non-AU – non action utterance; N – total number of pauses; PD, Parkinson Disease patients; SP – silent pause; T – total duration of pauses; WS – within subordinate; * indicate statistically significant differences.

S4 - Correlation analyses between pausing parameters differentiating the two groups, cognitive abilities and motor symptoms in PD patients.

	SWCT- IE-T		***SW-T***		***CL-T***		***WCST- PE***		***Ac-N***		***Ob-N***		***MDS-UPDRS-III***	
	**ρ**	**p**	**ρ**	**p**	**ρ**	**p**	**ρ**	**p**	**ρ**	**p**	**ρ**	**p**	**ρ**	**p**
Cookie Theft														
FP-Baa-AU-N	0.03	0.88	0.21	0.27	0.21	0.28	-0.17	0.37	0.10	0.63	0.28	0.16	-0.13	0.52
FP-Baa-AU-t	0.02	0.93	0.22	0.25	0.22	0.26	-0.19	0.32	0.10	0.62	0.31	0.12	-0.13	0.52
FP-WS-AU-t	0.03	0.87	-0.01	0.96	-0.01	0.98	-0.10	0.62	0.05	0.79	0.03	0.87	0.24	0.21
Quarrel														
SP-DURMean	0.20	0.29	-0.22	0.25	-0.24	0.22	0.17	0.37	-0.30	0.13	-0.48	0.01*	0.48	0.008*
SP-Baa-non-AU-t	0.52	0.004*	-0.52	0.004*	-0.54	0.003*	0.25	0.19	-0.54	**0.004****	-0.35	0.08	0.03	0.88

Legend: Ac-N, Action Naming; AU – action utterance; Baa – before other parts of speech; CL-T -Clustering total score; FP – filled pause; MDS-UPDRS-III scale, Movement Disorder Society-Sponsored Revision of the Unified Parkinson's Disease Rating Scale Part III motor function; N – total number of pauses; non-AU – non-action utterance; Ob-N, Object Naming; SP – silent pause; SP-DURMean - Mean duration of silent pauses; SW-T; total number of switches in fluency tasks; SWCT-IE-T, Stroop Word-Color Test interference effect time; t – total duration of pauses; WCST-PE, Wisconsin Card Sorting Test perseverative errors; WS – within subordinate;.**Uncorrected significance (correlation surviving after Bonferroni correction, highlighted in bold); *Uncorrected significance (correlation not surviving after Bonferroni correction)
